# Systematic Review and Meta-Analysis of the Relationship between *EPHX1* Polymorphisms and Colorectal Cancer Risk

**DOI:** 10.1371/journal.pone.0043821

**Published:** 2012-08-23

**Authors:** Fei Liu, Ding Yuan, Yonggang Wei, Wentao Wang, Lvnan Yan, Tianfu Wen, Mingqing Xu, Jiayin Yang, Bo Li

**Affiliations:** Division of Liver Transplantation, Department of Liver and Vascular Surgery, West China Hospital, Sichuan University, Chengdu, Sichuan Province, China; Ohio State University Medical Center, United States of America

## Abstract

**Background:**

Microsomal epoxide hydrolase (EPHX1) plays an important role in both the activation and detoxification of PAHs, which are carcinogens found in cooked meat and tobacco smoking. Polymorphisms at exons 3 and 4 of the *EPHX1* gene have been reported to be associated with variations in EPHX1 activity. The aim of this study is to quantitatively summarize the relationship between *EPHX1* polymorphisms and colorectal cancer (CRC) risk.

**Methods:**

Two investigators independently searched the Medline, Embase, CNKI, and Chinese Biomedicine Databases for studies published before June 2012. Summary odds ratios (ORs) and 95% confidence intervals (CIs) for *EPHX1* Tyr113His (rs1051740) and His139Arg (rs2234922) polymorphisms and CRC were calculated in a fixed-effects model and a random-effects model when appropriate.

**Results:**

This meta-analysis yielded 14 case-control studies, which included 13 studies for Tyr113His (6395 cases and 7893 controls) and 13 studies for His139Arg polymorphisms (5375 cases and 6962 controls). Overall, the pooled results indicated that *EPHX1* Tyr113His polymorphism was not associated with CRC risk; while the His139Arg polymorphism was significantly associated with decreased CRC risk (Arg/His vs. His/His, OR = 0.90, 95%CI = 0.83–0.98; dominant model, OR = 0.92, 95%CI = 0.85–0.99). The statistically significant association between *EPHX1* His139Arg polymorphism and CRC was observed among Caucasians and population-based case-control studies. This association showed little heterogeneity and remained consistently strong when analyses were limited to studies in which genotype frequencies were in Hardy–Weinberg equilibrium, or limited to studies with matched controls. When cumulative meta-analyses of the two associations were conducted by studies’ publication time, the results were persistent and robust.

**Conclusion:**

This meta-analysis suggests that *EPHX1* Tyr113His polymorphism may be not associated with CRC development; while the *EPHX1* His139Arg polymorphism may have a potential protective effect on CRC.

## Introduction

Colorectal cancer (CRC) is the third most commonly diagnosed cancer in males and the second in females worldwide, with over 1.2 million new cancer cases and 608,700 deaths estimated to have occurred in 2008 [1]. In the United States, CRC is the third most common cancer and third leading cause of cancer death for both men and women [2]. In Europe, CRC represents one of the primary causes of cancer deaths [3] and in Asia, CRC is the fourth leading cause of mortality by cancer, and its incidence is increasing [4]. In recent years, the incidence of CRC is increasing in China, which accounts for about 6.5% of total cancers in urban areas and 4.6% in rural areas [5]. However, the mechanism of colorectal carcinogenesis is still not fully understood. As with other complex diseases, CRC is caused by both genetic and environmental factors [6]. Because well-recognized genetic predisposition syndromes account for less than 3% of CRC, low-penetrance genetic factors alone or in combination with environmental factors probably contribute to CRC development [7].

Red meat consumption has frequently shown an association with an increased risk of CRC. It has been proposed that this risk may be due to carcinogenic polycyclic aromatic hydrocarbons (PAHs) and heterocyclic amines produced when meat is cooked at high temperatures [8]. Microsomal epoxide hydrolase (mEH) (EPHX1) is an enzyme found on the endoplasmic reticulum of many tissues and is responsible for the hydrolysis of various epoxides, including PAHs [9]. Epoxides are often the most toxicologically active form of a drug or environmental chemical, because they are highly reactive oxidative metabolites. EPHX1 breaks the three-membered epoxide ring structure by the transaddition of water to form a less-reactive diol that can be conjugated and more readily excreted. Nevertheless, EPHX1 plays a dual role in the detoxification and activation of procarcinogens, and its role in carcinogenesis may depend on exposures to different environmental substrates [10].

The human *EPHX1* gene is 35.48 kb with nine exons and eight introns on chromosome 1q42.1. There are more than 110 validated single nucleotide polymorphisms (SNPs) in *EPHX1* gene reported in the dbSNP database (http://www.ncbi.nlm.nih.gov/SNP), two of which are common and the two alleles of *EPHX1* in codons 113 (site T337C, amino acid change Tyr113His, dbSNP: rs1051740) and 139 (A415G, His139Arg, rs2234922) affect enzyme activity [11]. The tyrosine to histidine substitution in exon 3 (Tyr113His) of the *EPHX1* gene decreases *in vitro* enzyme activity by 40%, whereas the histidine to arginine substitution in exon 4 (His139Arg) increases *in vitro* enzyme activity by 25% [11]. Given the known differential effect of *EPHX1* alleles in the detoxification of procarcinogens, it has been proposed that the two functional polymorphisms may affect cancer risk.

Over the last two decades, a number of studies were conducted to investigate the association between *EPHX1* polymorphisms and CRC risk in different populations. However, the results of these studies are conflicting rather than conclusive. Until recently, few studies had been conducted to examine association between *EPHX1* Tyr113His and His139Arg polymorphism and CRC risk by the systematic review or meta-analysis. In order to derive a comprehensive estimation of the associations between *EPHX1* polymorphisms and CRC risk, we conducted a meta-analysis to assess the association between Tyr113His and His139Arg polymorphisms of the *EPHX1* gene and CRC susceptibility.

## Materials and Methods

### Literature Search Strategy

We searched the PubMed, Embase, CNKI (China National Knowledge Infrastructure) and Chinese Biomedicine databases for all articles on the association between *EPHX1* polymorphisms and CRC risk (last search update 5th June 2012). The following key words were used: “microsomal epoxide hydrolase” or “EPHX1” or “mEH”, “colorectal” or “colo*”, “cancer” or “tumor” or “carcinoma”, and “polymorphism” or “variant” or “allele” or “genotype”. The search was without restriction to the language and on studies conducted on human subjects. The reference lists of reviews and retrieved articles were hand searched at the same time. We did not consider abstracts or unpublished reports. If more than one article was published by the same author using the same case series, we selected the study where the most individuals were investigated.

### Inclusion and Exclusion Criteria

We reviewed abstracts of all citations and retrieved studies. The following criteria were used to include published studies: (i) case–control studies were conducted to evaluate the association between at least one of these two polymorphisms (Tyr113His and His139Arg) and CRC risk; (ii) sufficient genotype data were presented to calculate the odds ratios (ORs) and 95% confidence intervals (CIs); (iii) The paper should clearly describe CRC diagnoses and the sources of cases and controls. Major reasons for exclusion of studies were (i) review, or editorial, or comment; (ii) duplicated studies; (iii) cell line studies.

### Data Extraction

Two investigators (Fei Liu and Ding Yuan) extracted information from all eligible publications independently according to the inclusion criteria listed above. Disagreements were resolved by discussion between the two investigators. The following characteristics were collected from each study: the first author’s name, year of publication, the country of participants, ethnicity, source of control group (population- or hospital-based controls), number of cases and controls, genotypes, genotyping methods, minor allele frequency (MAF) in controls, and evidence of Hardy–Weinberg equilibrium (HWE) (Table 1). According to definitions in previous study [12], population-based case-control study (PCC) was defined as controls from healthy people, and hospital-based case-control study (HCC) were from hospitalized patients.

**Table 1:** Characteristics of studies included in this meta-analysis.

First author Reference	Year	Country	Ethnicity	SNPs studied	Source of Controls	Sample size (case/control)	Genotyping Methods	MAF in Controls	HWE
Harrison [21]	1999	UK	Caucasian	Tyr113His; His139Arg	PCC	101/203	PCR-RFLP	0.31; 0.15	0.04; 0.47
Sachse [22]	2002	UK	Caucasian	Tyr113His; His139Arg	PCC	490/593	PCR-RFLP	0.38; 0.19	0.00; 0.06
Yu [23]	2004	China	Asian	His139Arg	PCC	140/340	PCR-RFLP	0.10	0.37
Landi [24]	2005	Spain	Caucasian	Tyr113His; His139Arg	HCC	363/323; 361/321	ASO-PCR	0.29; 0.17	0.45; 0.40
Robien [25]	2005	USA	Mixed	Tyr113His; His139Arg	PCC	1593/1960	Taqman	0.29; 0.20	0.42; 0.15
Tranah [26]	2005	USA	Caucasian	Tyr113His; His139Arg	PCC	197/490	Taqman	0.32; 0.18	0.69; 0.83
Tranah1 [26]	2005	USA	Caucasian	Tyr113His; His139Arg	PCC	273/453	Taqman	0.28; 0.19	0.49; 0.83
Van der Logt [27]	2006	Netherlands	Caucasian	Tyr113His; His139Arg	PCC	365/391; 371/414	DCAS-PCR	0.29; 0.20	0.71; 0.72
Kiss [28]	2007	Hungary	Caucasian	Tyr113His; His139Arg	HCC	500/500	PCR-RFLP	0.28; 0.18	0.05; 0.05
Skjelbred [29]	2007	Norway	Caucasian	Tyr113His; His139Arg	PCC	102/299	Taqman PCR-RFLP	0.33; 0.21	0.91; 0.07
Hlavata [31]	2010	Czech	Caucasian	Tyr113His; His139Arg	HCC	495/495	Taqman	0.32; 0.23	0.75; 0.31
Cleary [7]	2010	Canada	Caucasian	Tyr113His	PCC	1163/1292	Taqman	0.30	0.87
Nisa [32]	2012	Japan	Asian	Tyr113His; His139Arg	PCC	685/778	Taqman; PCR-RFLP	0.44; 0.18	0.35; 0.41
Sahin [33]	2012	Turkey	Caucasian	Tyr113His; His139Arg	HCC	68/116	PCR-RFLP	0.35; 0.19	0.02; 0.01

**Abbreviations**: SNPs- single nucleotide polymorphisms; HCC, hospital-based case-control;PCC, population-based case-control;PCR-RFLP, polymerase chain reaction-restriction fragment length polymorphism; ASO-PCR, allele-specific oligonucleotide-polymerase chain reaction;DCAS-PCR, dual-colour allele-specific polymerase chain reaction; MAF, minor allele frequency; HWE, Hardy–Weinberg equilibrium.

### Statistical Analysis

We first assessed HWE in the controls for each study using goodness-of-fit test (*chi-square* or *Fisher’s exact* test) and a *P*<0.05 was considered as statistically significant. The strength of the association between CRC and the *EPHX1* Tyr113His and His139Arg polymorphisms were estimated using ORs, with the corresponding 95% CIs. In addition, Z-test was also used, and the *P* value <0.05 indicated statistical significance for the association. The crude ORs and 95%CIs were calculated by several comparisons. Taking *EPHX1* Tyr113His as an example: co-dominant model (His/His vs. Tyr/Tyr and Tyr/His vs. Tyr/Tyr), dominant model (His/His+Tyr/His vs. Tyr/Tyr) and recessive model (His/His vs. Tyr/His+Tyr/Tyr) respectively [13].

Both the Cochran’s Q statistic [14] to test for heterogeneity and the *I* ^2^ statistic to quantify the proportion of the total variation due to heterogeneity [15] were calculated. A *P* value of more than the nominal level of 0.10 for the Q statistic indicated a lack of heterogeneity across studies, allowing for the use of a fixed-effects model (the Mantel–Haenszel method) [16];otherwise, the random-effects model(the DerSimonian and Laird method) was used [17]. All meta-analyses are presented as forest plots that include ORs and 95% CIs for all individual studies, as well as the pooled estimator. Shaded figures provided for all ORs have dimension proportional to study weight. The Galbraith plot was used to detect the potential sources of heterogeneity [18]. Heterogeneity was also explored using subgroup analysis with ethnicity, study sample size (≥1000/<1000 subjects), matched control (Yes/No), HWE in controls (Yes/No) and source of controls (HCC/PCC).

Sensitivity analyses were performed to assess the stability of the results, namely, a single case-control study in this meta-analysis was omitted each time to reflect the influence of the individual data set to the pooled OR. Several methods were used to assess the potential publication bias. Visual inspection of funnel plot asymmetry was conducted. The Begg’s rank correlation method [19] and the Egger’s weighted regression method [20] were used to statistically assess publication bias (*P*<0.05 was considered statistically significant). All analyses were done using STATA software, version 11.0 (STATA Corp., College Station, TX, USA). All the *P* values were two-sided.

## Results

### Characteristics of Studies

**Figure 1:**
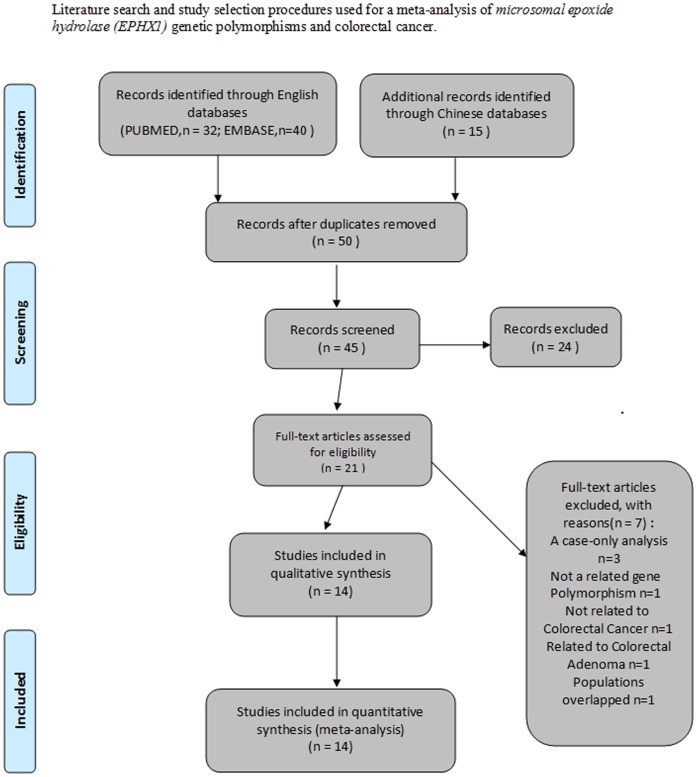
Literature search and study selection procedures used for a meta-analysis of *microsomal epoxide hydrolase (EPHX1)* genetic polymorphisms and colorectal cancer.

Through literature search and selection, a total of 15 case-control studies in 14 publications [7], [21]–[33], which included 14 studies for Tyr113His and 13 studies for His139Arg polymorphisms, were found to examine the *EPHX1* polymorphisms and CRC susceptibility. Because the populations in two studies [7], [30] were partially overlapped, we selected the study with the most individuals [7]. As a result, a total of 14 case-control studies in 13 publications [7], [21]–[29], [31]–[33], which included 13 studies for Tyr113His (6395 cases and 7893 controls) and 13 studies for His139Arg polymorphisms (5375 cases and 6962 controls), were identified based on MOOSE (Meta-analysis Of Observational Studies in Epidemiology) guidelines [34]. One article [26] mentioned two independent case-control studies (NHS and PHS), and the study was thus treated as two separate estimates. The literature search and study selection procedures are shown in Figure 1.

The characteristics of selected studies are summarized in Table 1. There were two studies of subjects of Asian descent, 11 studies of subjects of Caucasian descent and one of subjects Mixed descent. Studies had been carried out in China, UK, USA, Spain, Canada, Czech, Japan, Turkey, Norway, Hungary, and Netherlands. The cases definition used in the individual studies were pathologically or histologically diagnosed with CRC. Controls were mainly from healthy populations and matched for age and/or sex, of which 10 were population-based and four were hospital-based. Most of studies extracted DNA from peripheral blood and the classic PCR-RFLP assay and Taqman PCR were mainly used for genotyping. The genotype distributions among the controls of all studies followed HWE except for four studies [21], [22], [28], [33] for the Tyr113His polymorphism and one study [33] for the His139Arg polymorphism.

### Quantitative Synthesis

#### Association of the EPHX1 Tyr113His polymorphism with CRC susceptibility

**Figure 2:**
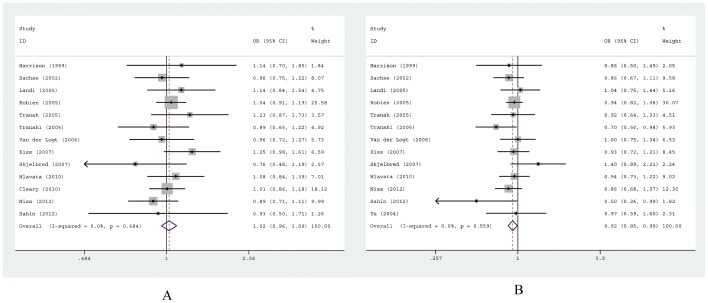
Forest plots of ORs with 95% CIs for *EPHX1* polymorphisms and risk for colorectal cancer. The center of each square represents the OR, the area of the square is the number of sample and thus the weight used in the meta-analysis, and the horizontal line indicates the 95%CI. (A) Tyr113His, His/His+Tyr/His vs. Tyr/Tyr. (B) His139Arg, Arg/Arg+Arg/His vs. His/His.

13 case-control studies [7], [21], [22], [24]–[29], [31]–[33] with 6395 cases and 7893 controls for *EPHX1* Tyr113His were included eventually. Table 2 listed the main results of this pooled analysis and Figure 2A showed the association of CRC risk with *EPHX1* Tyr113His polymorphism in the form of forest plots. Overall, the genotypes including at least one variant allele (His/His and Tyr/His) of the Tyr113His were not associated with CRC risk when compared with the wild-type Tyr/Tyr homozygote (His/His vs. Tyr/Tyr, OR = 1.08, 95%CI = 0.88–1.31; Tyr/His vs. Tyr/Tyr, OR = 1.03, 95%CI = 0.96–1.10). Similarly, no associations were observed in the dominant and recessive models (dominant model, OR = 1.02, 95%CI = 0.96–1.09; recessive model, OR = 1.08, 95%CI = 0.88–1.33).

**Table 2:** Quantitative analyses of the *EPHX1* Tyr113His polymorphism on the colorectal cancer (CRC) risk.

Genetic model			Homozygote		Heterozygote		Dominant model		Recessive model	
Variables	Sample size		His/His vs. Tyr/Tyr		Tyr/His vs. Tyr/Tyr		His/His+Tyr/His vs. Tyr/Tyr		His/His vs.Tyr/His+Tyr/Tyr	
	N^a^	Case/control	OR(95%CI)	*P_value_* b	OR(95%CI)	*P_value_* b	OR(95%CI)	*P_value_* b	OR(95%CI)	*P_value_* b
Total	13	6395/7893	1.08(0.88,1.31)	0.004	1.03(0.96,1.10)	0.704	1.02(0.96,1.09)	0.684	1.08(0.88,1.33)	<0.001
**Ethnicity**										
Caucasians	11	4117/5155	1.13(0.87,1.47)	0.002	1.04(0.95,1.14)	0.652	1.04(0.95,1.13)	0.678	1.14(0.86,1.50)	<0.001
Others	2	2278/2738	0.98(0.81,1.18)	0.217	1.00(0.89,1.13)	0.333	1.00(0.89,1.12)	0.224	0.99(0.83,1.18)	0.355
**Source of controls**										
HCC^c^	4	1426/1434	**1.33(1.02,1.73)**	0.117	1.11(0.95,1.29)	0.644	1.14(0.99,1.33)	0.770	1.36(0.88,2.08)	0.056
PCC^c^	9	4969/6459	0.98(0.79,1.21)	0.017	1.00(0.93,1.09)	0.640	0.99(0.92,1.07)	0.729	0.99(0.79,1.25)	0.003
**Study sample size**										
≥1000	5	4431/5123	0.99(0.76,1.29)	0.008	1.04(0.95,1.13)	0.626	1.02(0.94,1.11)	0.343	0.97(0.76,1.25)	0.006
<1000	8	1964/2770	1.18(0.87,1.62)	0.054	1.00(0.89,1.14)	0.513	1.03(0.91,1.16)	0.695	1.22(0.87,1.72)	0.011
**Matched control**										
Yes	8	3871/4717	1.00(0.78,1.27)	0.019	1.04(0.95,1.14)	0.583	1.02(0.93,1.11)	0.475	0.99(0.77,1.26)	0.008
No	5	2524/3176	1.24(0.86,1.78)	0.043	1.00(0.90,1.12)	0.545	1.03(0.93,1.14)	0.621	1.28(0.87,1.90)	0.016
**HWE** d **in controls**										
Yes	9	5236/6481	0.98(0.86,1.11)	0.843	1.01(0.94,1.10)	0.671	1.01(0.94,1.09)	0.650	0.98(0.86,1.10)	0.902
No	4	1159/1412	1.73(0.77,3.90)	<0.001	1.08(0.91,1.28)	0.432	1.09(0.93,1.27)	0.463	1.79(0.75,4.28)	<0.001

a Number of comparisons.

b *P* value of Q-test for heterogeneity test. Random-effects model was used when *P* value for heterogeneity test <0.05; otherwise, fixed-effects model was used.

c HCC, hospital-based case-control; PCC, population-based case-control.

d HWE, Hardy–Weinberg equilibrium.

On the basis of the potential underestimation of the true effect of the polymorphism on the CRC risk, we stratified these studies according to ethnicity, source of controls, study sample size, matched control, and HWE in controls. Different ethnicities were categorized as Caucasians and others; while different source of controls were defined as HCC and PCC. In stratified analyses, the variant genotypes (His/His and Tyr/His) had no significant relationship with CRC in all of the subgroups except that a significantly increased CRC risk was observed among the HCC populations in the homozygote comparison. Also, no significant associations were found in the dominant and recessive models in any subgroup (Table 2).

#### Association of the EPHX1 His139Arg polymorphism with CRC susceptibility

13 case-control studies [21]–[29], [31]–[33] with 5375 cases and 6962 controls for *EPHX1* His139Arg were included eventually. Table 3 listed the main results of this pooled analysis and Figure 2B showed the association of CRC risk with *EPHX1* His139Arg polymorphism in the form of forest plots. Overall, the results of combined analyses of all studies suggested that the His139Arg polymorphism was significantly associated with decreased CRC risk (Arg/His vs. His/His, OR = 0.90, 95% I = 0.83–0.98; dominant model, OR = 0.92, 95% I = 0.85–0.99), without any between-study heterogeneity. However, the association was not observed in the homozygote comparison and recessive genetic models (homozygote comparison model, OR = 1.14, 95% CI = 0.86–1.52; recessive model, OR = 1.18, 95% CI = 0.89–1.57).

**Table 3:** Quantitative analyses of the *EPHX1* His139Arg polymorphism on the colorectal cancer (CRC) risk.

Genetic model			Homozygote		Heterozygote		Dominant model		Recessive model	
Variables	Sample size		Arg/Arg vs. His/His		Arg/His vs. His/His		Arg/Arg+Arg/His vs. His/His		Arg/Arg vs.Arg/His+His/His	
	N^a^	Case/control	OR(95%CI)	*P_value_* b	OR(95%CI)	*P_value_* b	OR(95%CI)	*P_value_* b	OR(95%CI)	*P_value_* b
Total	13	5375/6962	1.14(0.86,1.52)	0.067	**0.90(0.83,0.98)**	0.763	**0.92(0.85,0.99)**	0.559	1.18(0.89,1.57)	0.057
**Ethnicity**										
Caucasians	10	4949/6404	1.31(0.94,1.81)	0.166	**0.88(0.79,0.98)**	0.594	0.92(0.83,1.02)	0.344	1.35(0.98,1.88)	0.162
Asian	2	2278/2738	0.74(0.42,1.32)	0.224	0.89(0.72,1.10)	0.848	0.87(0.71,1.07)	0.643	0.91(0.33,2.47)	0.231
**Source of controls**										
HCC^c^	4	1426/1434	1.00(0.65,1.54)	0.586	0.92(0.79,1.08)	0.243	0.93(0.79,1.08)	0.302	1.03(0.67,1.57)	0.531
PCC^c^	9	5801/7708	1.22(0.84,1.78)	0.022	**0.90(0.82,0.98)**	0.857	0.92(0.84,1.00)	0.538	1.27(0.87,1.85)	0.020
**Study sample size**										
≥1000	4	5263/6372	0.86(0.67,1.12)	0.334	0.92(0.83,1.02)	0.737	0.91(0.82,1.00)	0.859	1.00(0.71,1.41)	0.287
<1000	9	1964/2770	1.41(0.99,2.02)	0.227	0.88(0.77,1.00)	0.558	0.93(0.82,1.05)	0.279	1.43(0.93,2.20)	0.234
**Matched control**										
Yes	7	4703/5966	0.92(0.68,1.24)	0.556	**0.85(0.76,0.96)**	0.460	**0.86(0.77,0.96)**	0.559	0.96(0.72,1.29)	0.497
No	6	2524/3176	1.53(0.91,2.58)	0.020	0.95(0.85,1.07)	0.971	0.98(0.88,1.09)	0.701	1.56(0.92,2.63)	0.017
**HWE** d **in controls**										
Yes	12	6068/7730	1.13(0.85,1.51)	0.055	**0.91(0.84,0.99)**	0.940	0.93(0.86,1.00)	0.753	1.17(0.88,1.56)	0.050
No	1	1159/1412	4.14(0.17,103.71)	NA^e^	**0.47(0.24,0.94)**	NA^e^	**0.50(0.26,0.99)**	NA^e^	5.18(0.21,128.89)	NA^e^

a Number of comparisons.

b *P* value of Q-test for heterogeneity test. Random-effects model was used when *P* value for heterogeneity test <0.1; otherwise, fixed-effects model was used.

c HCC, hospital-based case-control; PCC, population-based case-control.

d HWE, Hardy–Weinberg equilibrium.

e not applicable.

When stratifying by ethnicity and source of controls, the significantly decreased CRC risk was observed among Caucasians (Arg/His vs. His/His, OR = 0.88, 95%CI = 0.79–0.98) and PCC studies (Arg/His vs. His/His, OR = 0.90, 95%CI = 0.82–0.98). This association remained consistently strong when analyses were limited to studies in which genotype frequencies were in HWE (Arg/His vs. His/His, OR = 0.91, 95%CI = 0.84–0.99), or limited to studies with matched controls (Arg/His vs. His/His, OR = 0.85, 95%CI = 0.76–0.96; dominant model, OR = 0.86, 95%CI = 0.77–0.96). When stratifying by study sample size, this association was not observed neither among large sample studies (≥1000 subjects) nor among small sample studies (<1000 subjects) (Table 3).

### Heterogeneity Analysis

For Tyr113His polymorphism, there was substantial heterogeneity among these studies for homozygote comparison (His/His vs. Tyr/Tyr: *P*_heterogeneity_ = 0.004), and recessive model comparison (His/His vs.Tyr/His+Tyr/Tyr: *P*_heterogeneity_<0.001). For His139Arg polymorphism, mild between-study heterogeneity was also detected the homozygote comparison, and recessive model comparison. Galbraith plot analyses of all included studies were used to assess the potential sources of heterogeneity. Three studies [21], [22], [28] were found to be contributors of heterogeneity for Tyr113His polymorphism (Figure S1A). We re-evaluated the association after excluding these three outlier studies with reduced heterogeneity (His/His vs. Tyr/Tyr: *P*_heterogeneity_ = 0.614; His/His vs.Tyr/His+Tyr/Tyr: *P*_heterogeneity_ = 0.495). Only one study was found to be contributor of heterogeneity for His139Arg polymorphism (Figure S1B) and the heterogeneity was significant reduced when excluding the outlier study (Arg/Arg vs. His/His: *P*_heterogeneity_ = 0.521; Arg/Arg vs.Arg/His+His/His: *P*_heterogeneity_ = 0.212).

### Sensitivity Analysis

In the sensitivity analysis, the influence of each study on the pooled OR was examined by repeating the meta-analysis while omitting each study, one at a time. As for the association of the *EPHX1* Tyr113His with CRC risk, the study that had the most influence on the overall pooled estimates (Figure S2A) seemed to be the one conducted by Kiss *et al*. [28]; however, the sensitivity analysis showed that the ORs were 1.02 (95% CI: 0.96, 1.09) and 1.01 (95% CI: 0.94, 1.08) before and after the removal of that study, respectively, indicating high stability of the results. Because there is known methodological issue with PCR-RFLP analysis of Tyr113His SNP [27], we performed analysis without studies using the biased method. When excluding the studies using PCR-RFLP analysis of Tyr113His SNP, the estimated pooled OR still did not change at all (Table S1). As for the association of the *EPHX1* His139Arg with CRC risk, the study that had the most influence on the overall pooled estimates (Figure S2B) seemed to be the one conducted by Robien *et al*. [25]; however, the sensitivity analysis showed that the ORs were 0.92 (95% CI: 0.85, 0.99) and 0.91 (95% CI: 0.83, 0.99) before and after the removal of that study, respectively, indicating high stability of the results. When excluding the studies that were not in HWE, the estimated pooled OR still did not change at all (Table 2 and Table 3). This procedure proved that our results were reliable and robust.

### Cumulative Meta-analysis

Cumulative meta-analyses of the 2 associations were also conducted via the assortment of studies by publication time. Figure S3A shows results from the cumulative meta-analysis of the association of the *EPHX1* Tyr113His with overall CRC in chronologic order. Inclinations toward null significant associations were evident with each accumulation of more data over time. Figure S3B shows results from the cumulative meta-analysis of the association of the *EPHX1* His139Arg with overall CRC in chronologic order. Inclinations toward decreased significant associations were evident with each accumulation of more data over time, although associations were initially null.

### Publication Bias

Funnel plot, Begg’s and Egger’s tests were performed to evaluate publication bias of the literature on CRC. Figure S4 displayed funnel plots that examined the *EPHX1* polymorphisms and overall CRC risk included in the meta-analysis in dominant comparison model. The shape of funnel plots did not reveal any evidence of funnel plot asymmetry. The statistical results still did not show publication bias [(1) *EPHX1*Tyr113His, His/His vs. Tyr/Tyr: Begg’s test *P* = 0.50, Egger’s test *P* = 0.16; Tyr/His vs. Tyr/Tyr: Begg’s test *P* = 0.43, Egger’s test *P* = 0.33; dominant model: Begg’s test *P* = 1.00, Egger’s test *P* = 0.80; recessive model: Begg’s test *P* = 0.43, Egger’s test *P* = 0.12. (2) *EPHX1* His139Arg, Arg/Arg vs. His/His: Begg’s test *P* = 0.50, Egger’s test *P* = 0.23; Arg/His vs. His/His: Begg’s test *P* = 0.30, Egger’s test *P* = 0.12; dominant model: Begg’s test *P* = 0.86, Egger’s test *P* = 0.65; recessive model: Begg’s test *P* = 0.50, Egger’s test *P* = 0.21].

## Discussion

The present meta-analysis, including 14 case–control studies, explored the association between the Tyr113His and His139Arg polymorphisms of the *EPHX1* gene and CRC risk. We found that *EPHX1* Tyr113His polymorphism was not associated with CRC risk (6395 cases and 7893 controls). When subgroup analyses were performed by ethnicity, source of controls, study sample size, matched control, and HWE in controls; significant association was still not observed in any subgroup except for among hospital-based studies. Nevertheless, we found that *EPHX1* His139Arg polymorphism was associated with decreased CRC risk. When stratifying by ethnicity and source of controls, the significant association was observed among Caucasians and among PCC studies. Moreover, this association showed little heterogeneity (*I^2^* = 0) and remained consistently strong when analyses were limited to studies in which genotype frequencies were in HWE, or limited to studies with matched controls. When cumulative meta-analyses of the two associations were conducted by studies’ publication date, the results were persistent and robust.

EPHX1 is a critical enzyme in xenobiotic metabolism [35], which plays an important role in both the activation and detoxification of PAHs and aromatic amines. EPHX1 catalyzes the hydrolysis of arene, alkene, and aliphatic epoxides from PAHs and aromatic amines. This hydrolysis is generally a detoxification reaction because less reactive and more water-soluble trans-dihydrodiols are produced [36]. In a sense, EPHX1 is a protective enzyme involved in general oxidative defenses against a number of environmental substances, and its genetic polymorphisms, *EPHX1* Tyr113His and His139Arg, may affect enzyme activity [11]. Previous *in vitro* study found that *EPHX1* Tyr113His was associated with 40% of decreased enzyme activity, while His139Arg was associated with 25% of increased enzyme activity [11]. Based on the assumption that the Tyr allele at exon 3 and the His allele at exon 4 confer normal activity, whereas the His allele at exon 3 confers low activity and the Arg allele at exon 4 confers high activity, Benhamou *et al* [37] classified predicted EPHX1 activity as low (113HisHis/139HisHis, 113TyrHis/139HisHis and 113HisHis/139HisArg), intermediate (113 TyrTyr/139HisHis, 113 HisHis/139ArgArg and 113TyrHis/139HisArg) or high (113TyrTyr/139ArgArg, 113TyrTyr/139 HisArg and 113TyrHis/139ArgArg) on the presence or absence of the 2 polymorphisms. Similarly, Smith and Harrison [38]classified predicted EPHX1 activity as rapid (113 TyrTyr/139 HisArg or 113 TyrTyr/139 ArgArg); normal (113 TyrTyr/139 HisHis or 113 TyrHis/139 HisArg); slow (113 TyrHis/139 HisHis or 113 TyrHis/139 ArgArg); and very slow (113 HisHis/139 HisHis). Given the different enzyme (the EPHX1 protein) activity which depends on the polymorphic form, it is biologically plausible that the *EPHX1* His139Arg polymorphism may decrease the risk of CRC.

Interestingly, we found that the *EPHX1* His139Arg heterozygotes, but not the homozygotes, had a significantly decreased risk of CRC. The observed effect is due mostly to the presence of heterozygous genotype and homozygous variant genotype rather dilutes this effect (Table 3 - heterozygous vs. dominant model). From the functional view there is lack of dose-relationship where the highest activity should exert the most significant effect. Although the reason for a significantly decreased risk associated with the His139Arg variant heterozygote remains unknown, it is possible that these heterozygotes may have impaired function because of the potential imbalance of the protein structure. Another possible explanation is that the heterozygous genotype may be in linkage disequilibrium with other susceptibility loci. Similar phenomenon was observed by Ma *et al.* [39], who studied the variant genotypes of CDKN1A and CDKN1B and breast cancer risk. They found that the CDKN1B C -79T heterozygotes, but not the homozygotes, had a significantly increased risk of breast cancer.

Our results were in part consistent with previous studies. For example, Li *et al.* [40] performed a comprehensive meta-analysis of published epidemiological studies aims to systematically evaluate putative EPHX1 enzyme activity and risk of cancers and found that putative EPHX1 enzyme activity is related with risk of lung and upper aerodigestive tract cancers. However, they did not find any association between *EPHX1* Tyr113His and His139Arg polymorphism and CRC risk. In recent, Zhao *et al* [41] published a meta-analysis for the relationships between five metabolic gene (including EPHX1) polymorphisms and colorectal adenoma risk and found that *EPHX1* Tyr113His and His139Arg did not have any associations with colorectal adenoma risk. Although the reasons for this difference are as yet unknown, some possibilities should be considered. First, those gene–variant associations vary in different kinds of diseases and may result from the different mechanisms of carcinogenesis among different kinds of tumor. Second, different ethnic composition may contribute to the discrepancy. Different meta-analyses included different original studies which were performed in different races and the ethnic composition in different meta-analyses may be diversity. Third, some methodological diversity, such as inclusion criteria, the quality of original studies, selection bias, Type I error and study sample size, also can contribute to the discrepancy.

Because the allele frequencies of polymorphisms and their effects on the cancer risk were diverse in the different ethnicities, we carried out subgroup analysis by ethnicity. The results demonstrated that *EPHX1* His139Arg polymorphism was associated with a decreased CRC risk among Caucasians, while there was no association between *EPHX1* His139Arg polymorphism and CRC risk among Asians. The null result in Asians may be due to the limited number of studies with only two studies from Asian available in this meta-analysis. It is critical that larger and well-designed multicentric studies based on Asian patients should be performed to re-evaluate the association. Moreover, results of meta-analyses often depend on control selection procedures [42]. Different controls source may be a confounding factor which may impact on the conclusion of our study because of case–control studies. For instance, some studies used a healthy population as the reference group (PCC), whereas others selected hospitalized patients as the reference group (HCC). In order to eliminate interference from the confounding factor, we performed subgroup analysis by source of controls. Our results showed that the significant association between *EPHX1* His139Arg polymorphism and CRC was observed among PCC, but not among HCC. This may be due to that the HCC studies have some selection biases because such controls might be ill-related population, and may not be a representative of the general population, especially when the investigated genotypes were associated with the disease conditions hospital-based controls might have. Although hospital controls are relatively easier, more convenient and economical to be recruited, a proper population-based control subject may be better to reduce biases in such genetic association studies.

One of the major concerns in a sound meta-analysis is the degree of heterogeneity that exists between the component studies because non-homogeneous data are liable to results in misleading results. In the present study, the Q-test and *I* ^2^ statistics were carried out to test the significance of heterogeneity. Obvious heterogeneity between studies was observed in overall comparisons and also some subgroup analyses. In an attempt to find the sources of heterogeneity, a Galbraith plot was drawn, and three studies were thought to serve as the main contributors for the Tyr113His polymorphism and only one study for the His139Arg polymorphism. The heterogeneity was significantly reduced when excluding the outlier studies. Moreover, we re-analyzed the association after excluding the outlier studies; the conclusion was still consistent in overall comparisons. Another important issue for any meta-analysis is publication bias due to selective publication of reports. In the current study, Begg’s funnel plot and Egger’s test were performed to evaluate this problem. Both the shape of funnel plots and statistical results did not show publication bias. It is worth mentioning that the results held when the sensitivity analysis was performed, which implied that the results were reliable.

Some limitations of this meta-analysis should be addressed. First, our meta-analysis was based on unadjusted OR estimates because not all published studies presented adjusted ORs or when they did, the ORs were not adjusted by the same potential confounders, such as age, sex, ethnicity and exposures. Lacking of the information for the data analysis may cause serious confounding bias. Second, this paper was limited by analyzing two single-SNPs respectively and lack of combination of two-SNP analysis. EPHX1 enzyme activity is affected by single or combination of polymorphisms Tyr113His and His139Arg [11], [43]. Based on the genotype combination of these two functional polymorphisms, Benhamou and colleagues [37] classified EPHX1 activity as putative low, intermediate and high. Thus, a meta-analysis that performed both single-SNP analysis and combined two-SNPs analysis may provide insights into the relationship between EPHX1 enzyme activity and CRC risk. However, only limited studies in this meta-analysis reported combination of two-SNP analyses (Table S2), which prevented us to perform pooled analysis. Third, there was significant between-study heterogeneity from studies of the *EPHX1* polymorphism, and the genotype distribution also showed deviation from HWE in some studies. In spite of these, our meta-analysis also had some advantages. First, we did not detect any publication bias indicating that the whole pooled result should be unbiased. Second, the quality of case–control studies included in current meta-analysis was satisfactory and met our inclusion criterion.

In conclusion, this meta-analysis evaluates the relationship between genetic polymorphisms and CRC risk and reveals that *EPHX1* Tyr113His polymorphism may be not associated with CRC development; while the *EPHX1* His139Arg polymorphism may have a potential protective effect on CRC. Since limited studies were from Asian populations, it is critical that larger and well-designed multicentric studies based on Asians should be performed to re-evaluate the association. Moreover, further studies estimating the effect of both single-SNP analysis and combination of two-SNP analysis and gene–environment interactions may eventually provide a better, comprehensive understanding of the association between the *EPHX1* polymorphisms and CRC risk.

## Supporting Information

Figure S1 **Galbraith plots for heterogeneity test of Tyr113His and His139Arg polymorphisms.** (A) Galbraith plot of the association between Tyr113His polymorphism and CRC risk (The studies outside the range between -2 and 2 were seen as the outliers and the major source of heterogeneity); (B) Galbraith plot of the correlation between His139Arg polymorphism and CRC risk.
(TIF)

Figure S2 **Influence analysis of the summary odds ratio coefficients on the association between** ***EPHX1*** **polymorphisms and colorectal cancer risk.** Results were computed by omitting each study (left column) in turn. Bars, 95% confidence interval. (A), For *EPHX1* Tyr113His His/His -plus-Tyr/His genotypes vs. Tyr/Tyr genotype; (B), For *EPHX1* His139Arg Arg/Arg-plus-Arg/His genotypes vs. His/His genotype.
(TIF)

Figure S3 **Results from cumulative meta-analysis of associations between** ***EPHX1*** **polymorphisms and colorectal cancer risk.** The circles and horizontal lines show the accumulation of estimates as results from each study were added, rather than the estimate for each individual study. Studies sorted by publication time; Bars, 95% confidence interval. (A), For *EPHX1* Tyr113His His/His-plus-Tyr/His genotypes vs. Tyr/Tyr genotype; (B), For *EPHX1* His139Arg Arg/Arg-plus-Arg/His genotypes vs. His/His genotype.
(TIF)

Figure S4 **Begg’s funnel plot for publication bias test. Each point represents a separate study for the indicated association.** LogOR, natural logarithm of OR. Horizontal line, mean effect size.(A), For *EPHX1* Tyr113His polymorphism; (B), For *EPHX1* His139Arg polymorphism.
(TIF)

Table S1 **Sensitivity analysis of the** ***EPHX1*** **Tyr113His polymorphism on the CRC risk by including and excluding the studies using PCR-RFLP analysis.**
(DOC)

Table S2 **Studies of Predicted** ***EPHX1*** **Activity and Risk of Colorectal Cancer.**
(DOC)

## References

[1] Jemal A, Bray F (2011) Center MM, Ferlay J, Ward E, et al (2011) Global cancer statistics.CA Cancer J Clin. 61: 69–90. 10.3322/caac.20107 21296855

[2] American Cancer Society (2010) Cancer facts and figures. Available: http://www.cancer.org/acs/groups/content/@nho/documents/document/acspc-024113.pdf.

[3] Ferlay J, Parkin DM, Steliarova-Foucher E (2010) Estimates of cancer incidence and mortality in Europe in 2008. Eur J Cancer 46: 765–781. 20116997 10.1016/j.ejca.2009.12.014

[4] Sung JJ, Lau JY, Goh KL, Leung WK (2005) Asia Pacific Working Group on Colorectal Cancer. Increasing incidence of colorectal cancer in Asia: implications for screening. Lancet Oncol 6: 871–876. 16257795 10.1016/S1470-2045(05)70422-8

[5] Zhao P, Dai M, Chen W, Li N (2010) Cancer trends in China. Jpn J Clin Oncol 40: 281–285. 20085904 10.1093/jjco/hyp187

[6] Lichtenstein P, Holm NV, Verkasalo PK, Iliadou A, Kaprio J, et al (2000) Environmental and heritable factors in the causation of cancer–analyses of cohorts of twins from Sweden, Denmark, and Finland. N Engl J Med 343: 78–85. 10891514 10.1056/NEJM200007133430201

[7] Cleary SP, Cotterchio M, Shi E, Gallinger S, Harper P (2010) Cigarette smoking, genetic variants in carcinogen-metabolizing enzymes, and colorectal cancer risk. Am J Epidemiol 172: 1000–1014. 20937634 10.1093/aje/kwq245 PMC2984254

[8] Norat T, Bingham S, Ferrari P, Slimani N, Jenab M, et al (2005) Meat, fish, and colorectal cancer risk: the European Prospective Investigation into cancer and nutrition. J Natl Cancer Inst 97: 906–916. 15956652 10.1093/jnci/dji164 PMC1913932

[9] Arand M, Cronin A, Adamska M, Oesch F (2005) Epoxide hydrolases: structure, function, mechanism, and assay. Methods Enzymol 400: 569–588. 16399371 10.1016/S0076-6879(05)00032-7

[10] Zhang JH, Jin X, Li Y, Wang R, Guo W, et al (2003) Epoxide hydrolase Tyr113His polymorphism is not associated with susceptibility to esophageal squamous cell carcinoma in population of North China. World J Gastroenterol 9: 2654–2657. 14669306 10.3748/wjg.v9.i12.2654 PMC4612025

[11] Hassett C, Aicher L, Sidhu JS, Omiecinski CJ (1994) Human microsomal epoxide hydrolase: genetic polymorphism and functional expression in vitro of amino acid variants. Hum Mol Genet 3: 421–428. 7516776 10.1093/hmg/3.3.421 PMC4868095

[12] Liu L, Liu L, Zeng F, Wang K, Huang J, et al (2011) Meta-analysis of the association between VEGF-634 G>C and risk of malignancy based on 23 case-control studies. J Cancer Res Clin Oncol 137: 1027–1036. 21174216 10.1007/s00432-010-0966-9 PMC11828216

[13] Attia J, Thakkinstian A, D’Este C (2003) Meta-analyses of molecular association studies: methodologic lessons for genetic epidemiology. J Clin Epidemiol 56: 297–303. 12767405 10.1016/s0895-4356(03)00011-8

[14] Cochran WG (1954) The combination of estimates from different experiments. Biometrics 10: 101–129.

[15] Higgins JP, Thompson SG, Deeks JJ, Altman DG (2003) Measuring inconsistency in meta- analyses. BMJ 327: 557–560. 12958120 10.1136/bmj.327.7414.557 PMC192859

[16] Mantel N, Haenszel W (1959) Statistical aspects of the analysis of data from retrospective studies of disease. J Natl Cancer Inst 22: 719–748. 13655060

[17] DerSimonian R, Laird N (1986) Meta-analysis in clinical trials.Control Clin Trials. 7: 177–188. 10.1016/0197-2456(86)90046-2 3802833

[18] Galbraith RF (1988) A note on graphical presentation of estimated odds ratios from several clinical trials. Stat Med 7: 889–894. 3413368 10.1002/sim.4780070807

[19] Begg CB, Mazumdar M (1994) Operating characteristics of a rank correlation test for publication bias. Biometrics 50: 1088–1101. 7786990

[20] Egger M, Davey Smith G, Schneider M, Minder C (1997) Bias in meta-analysis detected by a simple, graphical test. BMJ 315: 629–634. 9310563 10.1136/bmj.315.7109.629 PMC2127453

[21] Harrison DJ, Hubbard AL, MacMillan J, Wyllie AH, Smith CA (1999) Microsomal epoxide hydrolase gene polymorphism and susceptibility to colon cancer. Br J Cancer 79: 168–171. 10408710 10.1038/sj.bjc.6690028 PMC2362155

[22] Sachse C, Smith G, Wilkie MJ, Barrett JH, Waxman R, et al (2002) A pharmacogenetic study to investigate the role of dietary carcinogens in the etiology of colorectal cancer. Carcinogenesis 23: 1839–1849. 12419832 10.1093/carcin/23.11.1839

[23] Yu WP (2004) An epidemiological study on environmental exposure factors and genetic polymorphisms of colorectal cancer. Available at: http://dlib3.edu.cnki.net/kns50/detail.aspx?dbname = CMFD2004&filename = 2004062068.nh. [Article in Chinese].

[24] Landi S, Gemignani F, Moreno V, Gioia-Patricola L, Chabrier A, et al (2005) A comprehensive analysis of phase I and phase II metabolism gene polymorphisms and risk of colorectal cancer.Pharmacogenet Genomics. 15: 535–546. 10.1097/01.fpc.0000165904.48994.3d 16006997

[25] Robien K, Curtin K, Ulrich CM, Bigler J, Samowitz W, et al (2005) Microsomal epoxide hydrolase polymorphisms are not associated with colon cancer risk. Cancer Epidemiol Biomarkers Prev 14: 1350–1352. 15894702 10.1158/1055-9965.EPI-04-0877

[26] Tranah GJ, Chan AT, Giovannucci E, Ma J, Fuchs C, et al (2005) Epoxide hydrolase and CYP2C9 polymorphisms, cigarette smoking, and risk of colorectal carcinoma in the Nurses’ Health Study and the Physicians’ Health Study. Mol Carcinog 44: 21–30. 15924351 10.1002/mc.20112

[27] van der Logt EM, Bergevoet SM, Roelofs HM, Te Morsche RH, Dijk Y, et al (2006) Role of epoxide hydrolase, NAD(P)H:quinone oxidoreductase, cytochrome P450 2E1 or alcohol dehydrogenase genotypes in susceptibility to colorectal cancer.Mutat Res. 593: 39–49. 10.1016/j.mrfmmm.2005.06.018 16039674

[28] Kiss I, Orsós Z, Gombos K, Bogner B, Csejtei A, et al (2007) Association between allelic polymorphisms of metabolizing enzymes (CYP 1A1, CYP 1A2, CYP 2E1, mEH) and occurrence of colorectal cancer in Hungary. Anticancer Res 27: 2931–2937. 17695473

[29] Skjelbred CF, Saebø M, Hjartåker A, Grotmol T, Hansteen IL, et al (2007) Meat, vegetables and genetic polymorphisms and the risk of colorectal carcinomas and adenomas.BMC Cancer. 7: 228. 10.1186/1471-2407-7-228 PMC2228310 18093316

[30] Cotterchio M, Boucher BA, Manno M, Gallinger S, Okey AB, et al (2008) Red meat intake, doneness, polymorphisms in genes that encode carcinogen-metabolizing enzymes, and colorectal cancer risk.Cancer Epidemiol Biomarkers Prev. 17: 3098–3107. 10.1158/1055-9965.EPI-08-0341 PMC2751598 18990750

[31] Hlavata I, Vrana D, Smerhovsky Z, Pardini B, Naccarati A, et al (2010) Association between exposure-relevant polymorphisms in CYP1B1, EPHX1, NQO1, GSTM1, GSTP1 and GSTT1 and risk of colorectal cancer in a Czech population. Oncol Rep 24: 1347–1353. 20878130 10.3892/or_00000992

[32] Nisa H, Budhathoki S, Morita M, Toyomura K, Nagano J, et al (2012) Microsomal epoxide hydrolase polymorphisms, cigarette smoking, and risk of colorectal cancer: The Fukuoka Colorectal Cancer Study. Mol Carcinog. Doi: 10.1002/mc.21897. [Epub ahead of print]. 10.1002/mc.21897 22415791

[33] Sahin O, Arikan S, Oltulu YM, Coskunpinar E, Eren A, et al (2012) Investigation of a Possible Relationship Between EPHX1 Gene Polymorphisms and Colorectal Cancer in Turkish Society. Genet Test Mol Biomarkers 16: 423–428. 22352736 10.1089/gtmb.2011.0223

[34] Stroup DF, Berlin JA, Morton SC, Olkin I, Williamson GD, et al (2000) Meta-analysis of observational studies in epidemiology: a proposal for reporting. Meta-analysis Of Observational Studies in Epidemiology (MOOSE) group. JAMA 283: 2008–2012. 10789670 10.1001/jama.283.15.2008

[35] Omiecinski CJ, Hassett C, Hosagrahara V (2000) Epoxide hydrolase–polymorphism and role in toxicology. Toxicol Lett 112–113: 365–370. 10.1016/s0378-4274(99)00235-0 10720753

[36] Oesch F (1973) Mammalian epoxide hydrases: inducible enzymes catalysing the inactivation of carcinogenic and cytotoxic metabolites derived from aromatic and olefinic compounds. Xenobiotica 3: 305–340. 4584115 10.3109/00498257309151525

[37] Benhamou S, Reinikainen M, Bouchardy C, Dayer P, Hirvonen A (1998) Association between lung cancer and microsomal epoxide hydrolase genotypes. Cancer Res 58: 5291–5293. 9850050

[38] Smith CA, Harrison DJ (1997) Association between polymorphism in gene for microsomal epoxide hydrolase and susceptibility to emphysema. Lancet 350: 630–633. 9288046 10.1016/S0140-6736(96)08061-0

[39] Ma H, Jin G, Hu Z, Zhai X, Chen W, et al (2006) Variant genotypes of CDKN1A and CDKN1B are associated with an increased risk of breast cancer in Chinese women. Int J Cancer 119: 2173–2178. 16804901 10.1002/ijc.22094

[40] Li X, Hu Z, Qu X, Zhu J, Li L, et al (2011) Putative EPHX1 enzyme activity is related with risk of lung and upper aerodigestive tract cancers: a comprehensive meta-analysis. PLoS One 6: e14749. 21445251 10.1371/journal.pone.0014749 PMC3060809

[41] Zhao ZQ, Guan QK, Yang FY, Zhao P, Zhou B, et al (2012) System review and metaanalysis of the relationships between five metabolic gene polymorphisms and colorectal adenoma risk.Tumour Biol. 33: 523–535. 10.1007/s13277-011-0287-x 22161138

[42] Benhamou S, Lee WJ, Alexandrie AK, Boffetta P, Bouchardy C, et al (2002) Meta- and pooled analyses of the effects of glutathione S-transferase M1 polymorphisms and smoking on lung cancer risk. Carcinogenesis 23: 1343–1350. 12151353 10.1093/carcin/23.8.1343

[43] Hassett C, Lin J, Carty CL, Laurenzana EM, Omiecinski CJ (1997) Human hepatic microsomal epoxide hydrolase: comparative analysis of polymorphic expression. Arch Biochem Biophys 337: 275–283. 9016823 10.1006/abbi.1996.9794

