# Supplementary figures and images for: Systematic Review and Meta-Analysis of the Relationship between EPHX1 Polymorphisms and Colorectal Cancer Risk
Source: PLoS One. 2012 Aug 23;7(8):e43821. doi: 10.1371/journal.pone.0043821 (PMC3426545; doi:10.1371/journal.pone.0043821)

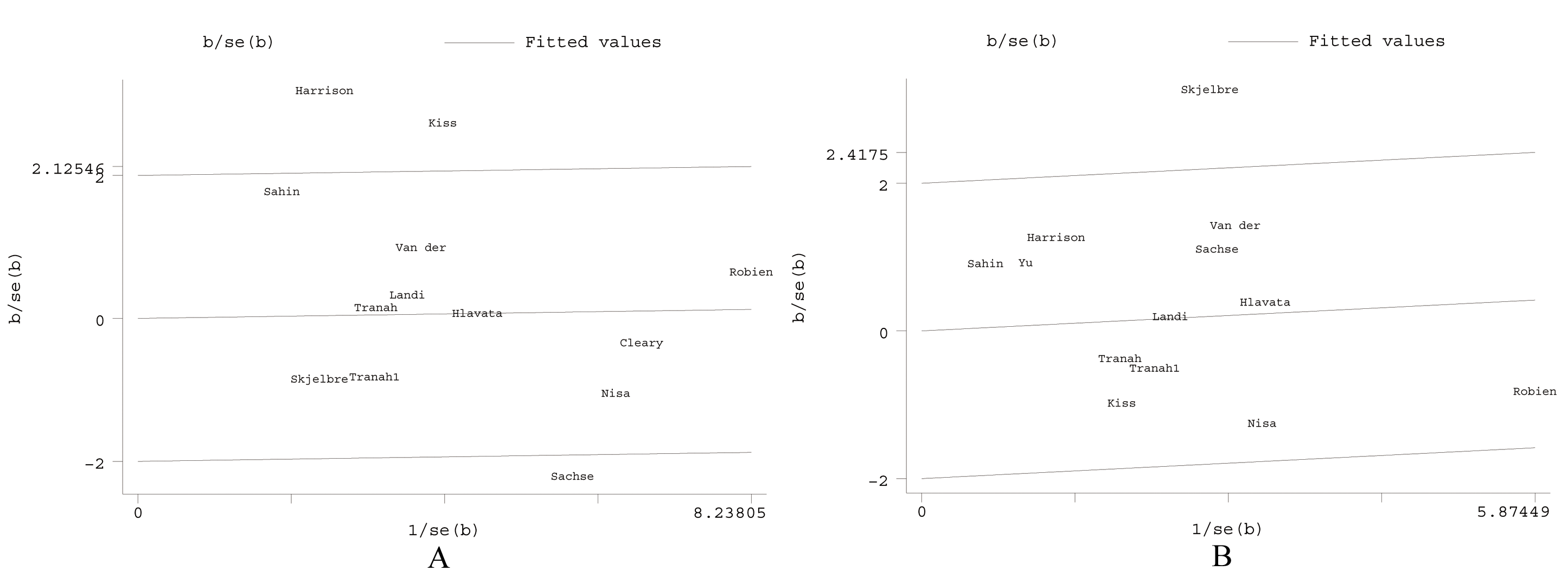

Supplement: Figure S1 — Galbraith plots for heterogeneity test of Tyr113His and His139Arg polymorphisms. (A) Galbraith plot of the association between Tyr113His polymorphism and CRC risk (The studies outside the range between -2 and 2 were seen as the outliers and the major source of heterogeneity); (B) Galbraith plot of the correlation between His139Arg polymorphism and CRC risk. (TIF) [file pone.0043821.s001.tif]

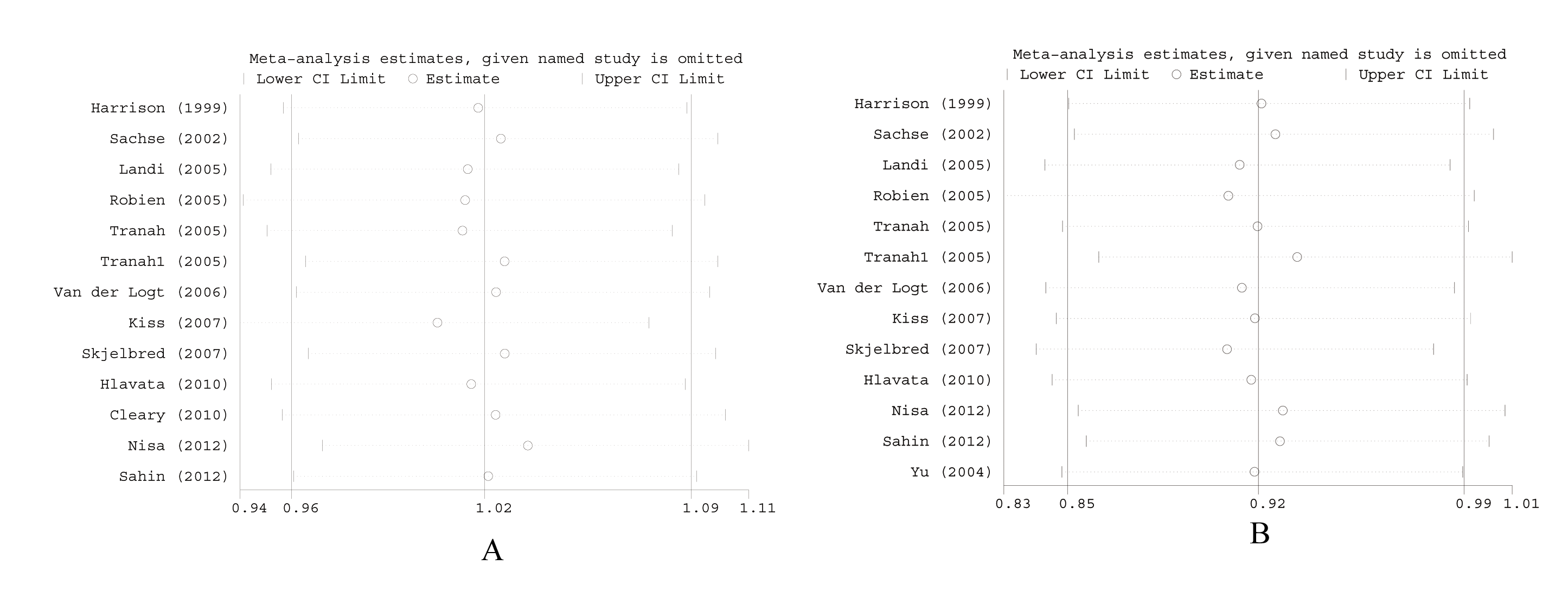

Supplement: Figure S2 — Influence analysis of the summary odds ratio coefficients on the association between EPHX1 polymorphisms and colorectal cancer risk. Results were computed by omitting each study (left column) in turn. Bars, 95% confidence interval. (A), For EPHX1 Tyr113His His/His -plus-Tyr/His genotypes vs. Tyr/Tyr genotype; (B), For EPHX1 His139Arg Arg/Arg-plus-Arg/His genotypes vs. His/His genotype. (TIF) [file pone.0043821.s002.tif]

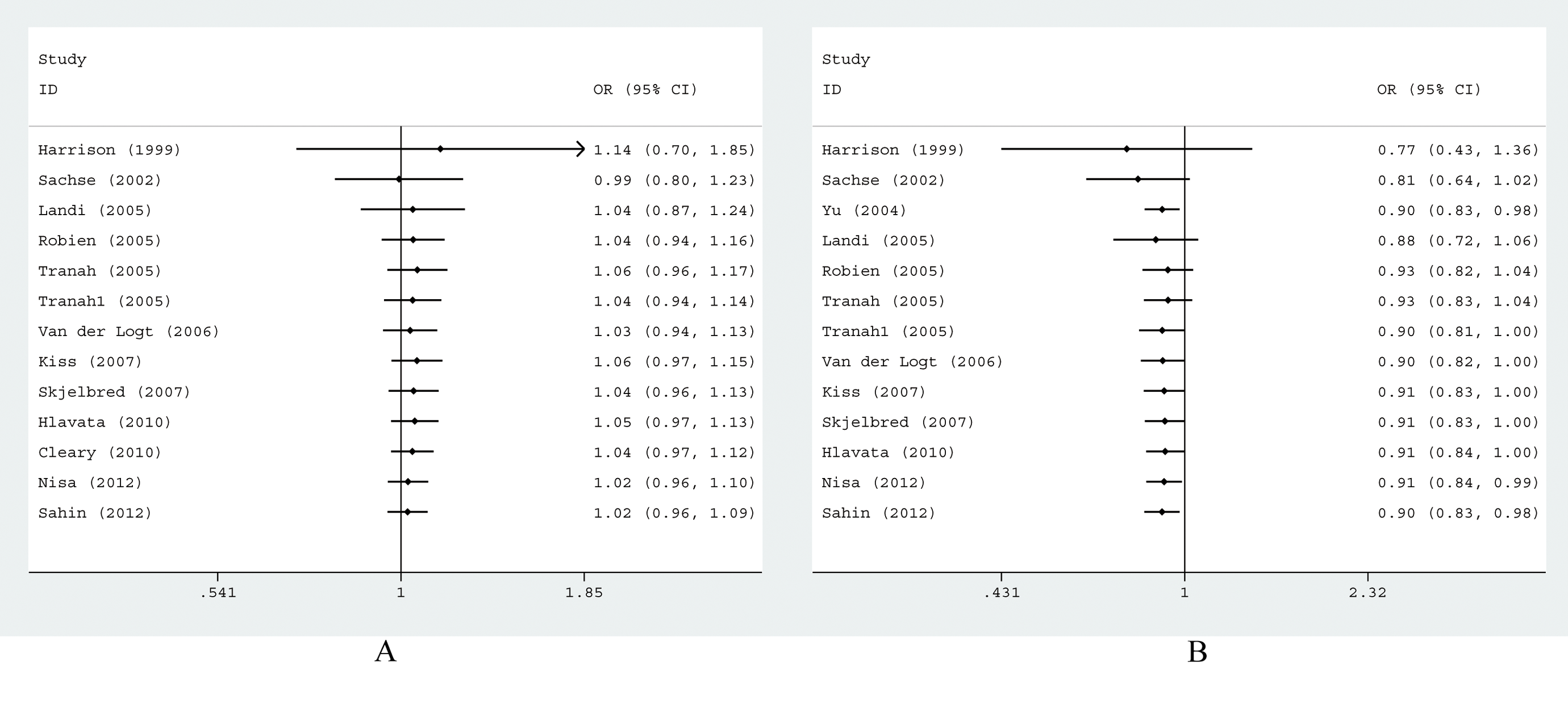

Supplement: Figure S3 — Results from cumulative meta-analysis of associations between EPHX1 polymorphisms and colorectal cancer risk. The circles and horizontal lines show the accumulation of estimates as results from each study were added, rather than the estimate for each individual study. Studies sorted by publication time; Bars, 95% confidence interval. (A), For EPHX1 Tyr113His His/His-plus-Tyr/His genotypes vs. Tyr/Tyr genotype; (B), For EPHX1 His139Arg Arg/Arg-plus-Arg/His genotypes vs. His/His genotype. (TIF) [file pone.0043821.s003.tif]

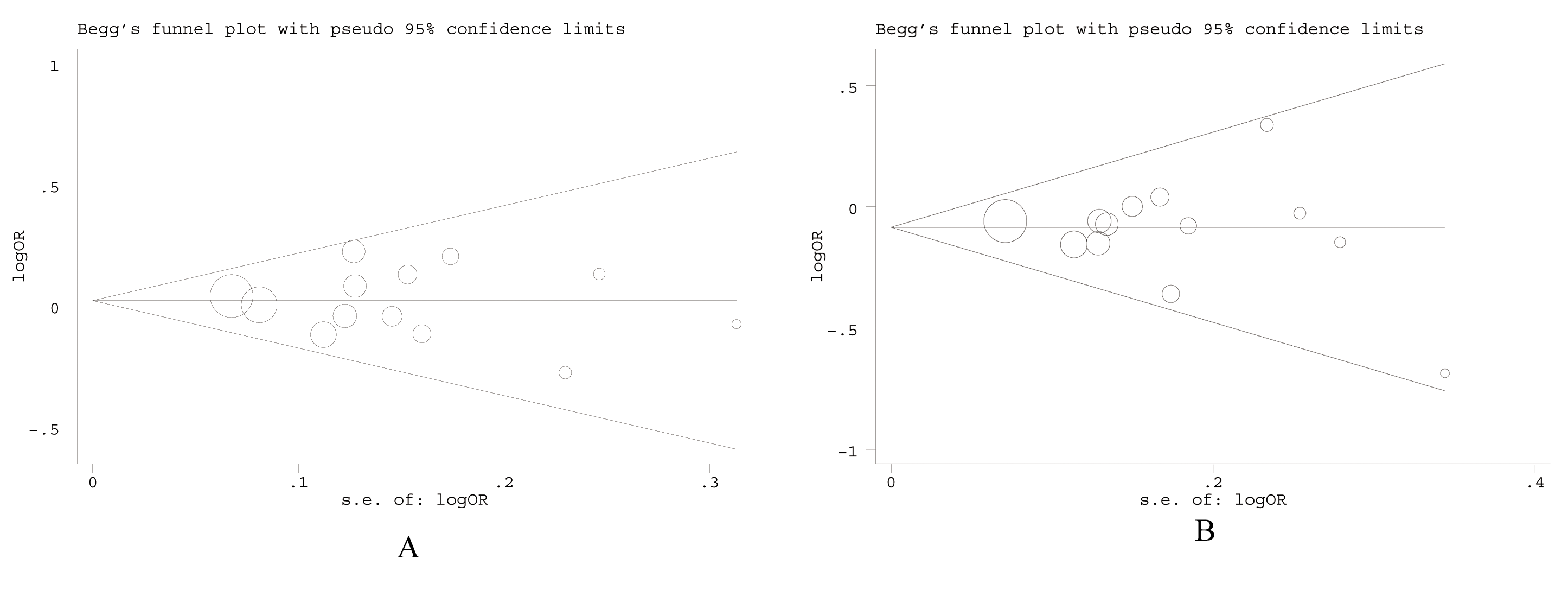

Supplement: Figure S4 — Begg’s funnel plot for publication bias test. Each point represents a separate study for the indicated association. LogOR, natural logarithm of OR. Horizontal line, mean effect size.(A), For EPHX1 Tyr113His polymorphism; (B), For EPHX1 His139Arg polymorphism. (TIF) [file pone.0043821.s004.tif]
