# Supplementary material for: Systematic Review and Meta-Analysis of the Relationship between EPHX1 Polymorphisms and Colorectal Cancer Risk
Source: PLoS One. 2012 Aug 23;7(8):e43821. doi: 10.1371/journal.pone.0043821 (PMC3426545; doi:10.1371/journal.pone.0043821)
Supplement: Table S1 — Sensitivity analysis of the EPHX1 Tyr113His polymorphism on the CRC risk by including and excluding the studies using PCR-RFLP analysis. (DOC) [file pone.0043821.s005.doc]

Table S1 . Sensitivity analysis of the *EPHX1* Tyr113His polymorphism on the CRC risk by including and excluding the studies using PCR-RFLP analysis.

| Genetic model | | | Homozygote | | Heterozygote | | Dominant model | | Recessive model | |
| --- | --- | --- | --- | --- | --- | --- | --- | --- | --- | --- |
| Variables | Sample size | | His/His vs. Tyr/Tyr | | Tyr/His vs. Tyr/Tyr | | His/His+Tyr/His vs. Tyr/Tyr | | His/His vs.Tyr/His+Tyr/Tyr | |
|  | N^a^ | Case/control | OR(95%CI) | *P_value_*^b^ | OR(95%CI) | *P_value_*^b^ | OR(95%CI) | *P_value_*^b^ | OR(95%CI) | *P_value_*^b^ |
| Total | 13 | 6395/7893 | 1.08(0.88,1.31) | 0.004 | 1.03(0.96,1.10) | 0.704 | 1.02(0.96,1.09) | 0.684 | 1.08(0.88,1.33) | ＜0.001 |
| **Genotyping method** | | | | | | | | | | |
| PCR-RFLP | 4 | 4117/5155 | 1.73(0.77,3.90) | ＜0.001 | 1.08(0.91,1.28) | 0.432 | 1.09(0.93,1.27) | 0.463 | 1.79(0.75,4.28) | ＜0.001 |
| Others | 9 | 2278/2738 | 0.98(0.86,1.11) | 0.843 | 1.01(0.94,1.10) | 0.671 | 1.01(0.94,1.09) | 0.650 | 0.98(0.86,1.10) | 0.902 |

a. Number of comparisons.

b. *P* value of Q-test for heterogeneity test. Random-effects model was used when *P* value for heterogeneity test <0.05; otherwise, ﬁxed-effects model was used.
