# Supplementary material for: Systematic Review and Meta-Analysis of the Relationship between EPHX1 Polymorphisms and Colorectal Cancer Risk
Source: PLoS One. 2012 Aug 23;7(8):e43821. doi: 10.1371/journal.pone.0043821 (PMC3426545; doi:10.1371/journal.pone.0043821)
Supplement: Table S2 — Studies of Predicted EPHX1 Activity and Risk of Colorectal Cancer. (DOC) [file pone.0043821.s006.doc]

Table S2. Studies of Predicted EPHX1 Activity and Risk of Colorectal Cancer

| Classiﬁcation based Benhamou et al. | | | | | | | | | | | |
| --- | --- | --- | --- | --- | --- | --- | --- | --- | --- | --- | --- |
| Citation | Slow* | | Intermediate | | Fast | | | Intermediate^a^ | | Fast^b^ | |
|  | n (cases/control) | | n (cases/control) | | n (cases/control) | | | OR (95% CI) | | OR (95% CI) | |
| Sachse | 197/246 | | 208/240 | | 84/106 | | | 1.08 (0.83–1.41) | | 0.99 (0.70–1.39) | |
| Nisa | 367/414 | | 223/246 | | 95/118 | | | 1.06 (0.84–1.33) | | 0.92 (0.67–1.25) | |
| Classiﬁcation based on Smith and Harrison. | | | | | | | | | | | |
| Citation | Rapid | Normal* | | Slow | | Very slow | Rapid^c^ | | Slow^d^ | | Very slow^e^ |
|  | n (cases/control) | | | | | | OR (95% CI) | | OR (95% CI) | | OR (95% CI) |
| Robien et al | 282/351 | 694/882 | | 468/553 | | 149/174 | 1.0 (0.8-1.2) | | 1.1 (0.9-1.2) | | 1.1 (0.8-1.4) |
| Tranah | 25/80 | 78/184 | | 59/135 | | 14/31 | 0.78(0.44–1.38) | | 1.00(0.63–1.57) | | 0.76(0.34–1.6) |
| Tranah1 | 36/78 | 123/174 | | 62/101 | | 12/29 | 0.62(0.39–1.00) | | 0.85(0.57–1.27) | | 0.58(0.28–1.2) |
| van der Logt | 67/63 | 160/178 | | 94/109 | | 25/14 | 1.5(0.86-2.6) | | 1.4 (0.86–2.4) | | 1.2 (0.73–1.9) |

*Reference group

a, OR (95% CI) for Intermediate vs. Slow

b, OR (95% CI) for Fast vs. Slow

c, OR (95% CI) for Rapid vs. Normal

d, OR (95% CI) for Slow vs. Normal

e, OR (95% CI) for Very slow vs. Normal
